# Trypanosomatids in Small Mammals of an Agroecosystem in Central Brazil: Another Piece in the Puzzle of Parasite Transmission in an Anthropogenic Landscape

**DOI:** 10.3390/pathogens8040190

**Published:** 2019-10-14

**Authors:** Elida M. V. Brandão, Samanta C. C. Xavier, Jeiel G. Carvalhaes, Paulo S. D’Andrea, Frederico G. Lemos, Fernanda C. Azevedo, Renata Cássia-Pires, Ana M. Jansen, André L. R. Roque

**Affiliations:** 1 Laboratório de Biologia de Tripanosomatídeos, Instituto Oswaldo Cruz, Fundação Oswaldo Cruz, Rio de Janeiro, RJ 21040900, Brasil; elida_millena@hotmail.com (E.M.V.B.); samanta@ioc.fiocruz.br (S.C.C.X.); jansen@ioc.fiocruz.br (A.M.J.); 2 Laboratório de Biologia e Parasitologia de Mamíferos Silvestres Reservatórios, Instituto Oswaldo Cruz, Fundação Oswaldo Cruz, Rio de Janeiro, RJ 21040900, Brasil; jgabrir@yahoo.com.br (J.G.C.); dandrea@ioc.fiocruz.br (P.S.D.); 3 Programa de Conservação Mamíferos do Cerrado (PCMC)–Unidade Acadêmica Especial de Biotecnologia, Universidade Federal de Goiás/Regional Catalão, Catalão, GO 75704020, Brasil; lemos.pcmc@gmail.com (F.G.L.); cavalcantifer@yahoo.com (F.C.A.); 4 Laboratório de Biologia de Parasitos, Centro de Ciências da Saúde, Departamento de Ciências Farmacêuticas, Universidade Federal do Rio Grande do Norte, Natal, RN 59012570, Brasil; renatacapires@gmail.com

**Keywords:** agroecosystems, Brazilian Cerrado, marsupials, rodents, canids, neotropical mammals, *Leishmania* sp., *Trypanosoma cruzi*

## Abstract

We surveyed infection by *Trypanosoma* spp. and *Leishmania* spp. in small wild mammals from Cumari, Goiás State aiming to investigate the diversity of trypanosomatid in a modified landscape of the Brazilian Cerrado (and possible infection overlapping with canids from the same area). Blood, skin, spleen, and liver samples were collected for parasitological, serological, and molecular assays. *Gracilinanus agilis* was the most abundant species (*N* = 70; 48.6%) and it was the only one with patent parasitemia. Characterization by mini-exon and 18SrDNA targets were achieved in 7/10 hemocultures with positive fresh blood examination, which confirmed the *T. cruzi* infection by Discrete Typing Units (DTU) TcI in single (*N* = 2) and mixed infections with other DTUs (*N* = 5). *T. rangeli* and *T. dionisii* were detected in skin fragments from *Didelphis albiventris* and *Oecomys cleberi,* respectively. *G. agilis* were found to be infected by *L. braziliensis* and *L. guyanensis,* while *Leishmania* sp. DNA was detected in the liver of *Oligoryzomys nigripes* and *Calomys expulsus*. Subpatent infection by *T. cruzi* and *Leishmania* sp. was serologically detected in 15% and 9% of the small mammal fauna, respectively. Small mammals from Cumari are included in *T. cruzi* and *Leshmania* spp. transmission cycles, showing a higher diversity of trypanosomatid species and/or genotypes than that observed in canids of the same agroecosystem.

## 1. Introduction

*Trypanosoma cruzi* and *Leishmania* spp. (Trypanosomatida; Trypanosomatidae) are enzootic parasites that are maintained in the Americas by dozens of species of mammals and transmitted by hematophagous vector insects, triatomines from the Reduviidae family, and female sandflies from *Lutzomyia* genus, respectively [1,2]. In humans, *T. cruzi* is the etiological agent of Chagas disease and six Discrete Typing Units (DTU) are currently recognized in this parasite: TcI to TcVI, besides Tcbat, a seventh DTU described in bats [3,4]. Despite proposed attempts to correlate *T. cruzi* subpopulations with host species, geographic distribution, and/or human disease, it has not yet been possible to detect any unequal association [5].

*Leishmania* spp. currently comprises more than 30 species, some of which are responsible for distinct clinical forms of human leishmaniasis as zoonotic diseases with high public health impact [6,7]. *Leishmania* species from mammals are divided into two subgenera: *L. (Leishmania)* and *L. (Viannia)*, which are grouped into eight monophyletic groups that correspond to so-called species complexes [8]. Seven of them: *L. donovani, L. mexicana, L. braziliensis, L. lainsoni, L. naiffi, L. lindenberg,* and *L. guyanensis* contain species already described as infecting humans in Brazil [9,10,11,12]. Although knowledge of leishmaniasis has improved in recent decades, little is known regarding the spectrum of host species (mammals and vectors) of this parasite in nature [13].

The Cerrado is the second largest Brazilian ecosystem and the second richest savannah biome of the world, containing several endemic species [14,15]; however, it has been suffering human interventions as a result of increasing deforestation for agricultural and livestock occupation [16,17,18]. The destruction of ecosystems leads to habitat decline, food restriction, and, in some cases, species extinction, leading populations of wild mammals to areas that bring them into contact with humans and domestic animals [19]. It is known that canids may make long displacements, through different types of habitats, such as open areas, forests, and remnants, where small mammals are present and can serve as a food source for them [20,21]. Canids may also act as bio-accumulators of parasites, since they are top chain predators [5], and these factors favor the spread of parasites in the environment.

It is in this scenario that the Limoeiro region (Cumari Municipality, Goiás, Brazil) is located, an area of Cerrado quite anthropically altered, where the Mammals from Cerrado Conservation Program (PCMC) develops conservation actions that are focused on endangered species of mammals. In this area, wild canids, such as the hoary fox (*Lycalopex vetulus*), crab-eating foxes (*Cerdocyon thous*), and maned wolves (*Chrysocyon brachyurus*), share the same areas with domestic species (e.g., dogs *Canis lupus familiaris* and cattle *Bos indicus*) and humans. Thus, PCMC has been joining multidisciplinary efforts to study aspects, such as ecology, genetics, and health of these animals. As a result of this work, infection by *T. cruzi* and *Leishmania* spp. in these wild canids has been diagnosed through serological assays, besides the isolation of *T. cruzi* DTU TcIII in hoary foxes [22,23]. Aiming to investigate the diversity of trypanosomatid species in the area (and possible infection overlaps among hosts from distinct taxa, as small mammals and canids), we surveyed the infection by *Trypanosoma* spp. and *Leishmania* sp. in the small mammal fauna and discuss its association with wild canids that are infected in the same area. We demonstrated a higher diversity of trypanosomatid species and/or genotypes of *T. cruzi* in small mammals than that observed in canids. We concluded that small mammals from Cumari are immersed in the transmission cycles of *T. cruzi* and *Leshmania* spp. and share, at least, one *T. cruzi* DTU (TcIII) with canids from the same area, showing overlapping transmission cycle among wild canids and small mammals.

## 2. Results

### 2.1. Small Mammal Fauna Composition

One hundred and forty-four small mammals were captured during the four expeditions between 2013 and 2015, totaling a capture success of 2.8% per trap/night. The number of captured specimens was higher in the dry season (*n* = 106; 73.6%) than in the wet season (*n* = 38; 26.4%) (*p* = 0.0001). The abundance of species of marsupials and rodents was similar (*p* > 0.05), with 74 marsupials (51.4%) and 70 rodents (48.6%) being captured. A greater richness of rodents than marsupials was registered for dry seasons (*p* = 0.016), whereas no difference was detected for wet seasons. The marsupial *Gracilinanus agilis* was the most abundant species to be captured (*n* = 70; 48.6%) (Table 1).

The ratio of sampling effort in the understory and ground strata was 1600 and 3600, respectively, corresponding to 80 understory traps and 180 ground traps for five nights each excursion (*N* = 4). Capture success was 79 small mammals captured in the understory (5%) and 65 captured in the ground stratum (1.8%). Species, such as *G. agilis, D. albiventris, O. cleberi, R. macrurus,* and *C. tener* were also captured in the understory. Thus, out of a total of 106 animals captured of these species, 79 were collected in the understory stratum, which represented 74.5% of the captured of these species.

### 2.2. Trypanosomatid Infection

One hundred and forty-two specimens were examined by fresh blood examination, and 10 (7%) of them (all *G. agilis*) were positive for the presence of flagellates. Of 233 hemocultures that were obtained from 129 small mammals, only one was positive, but it was not established. This culture was derived from one *G. agilis* also positive in fresh blood examination.

We successfully characterized, by the Mini exon assay, the sediment from seven cultures of the positive samples in fresh blood examination. These were characterized as single infection by *T. cruzi* TCI (*N* = 3) or mixed infection TCI/Z3 (Zymodeme 3) (*N* = 4). The four-mixed infection samples TCI/Z3 were submitted to PCR-RFLP of H3/AluI and two of them were characterized as DTU TcIV (Table 2).

The 18SrDNA nested PCR in all seven samples confirmed *Trypanosoma cruzi* infection. Of three samples that were characterized as TcI by the mini-exon assay, two confirmed the same DTU and one was identified as TcII, which suggested a mixed infection. Two samples that were suggestive of *T. cruzi* TcIV were previously characterized as mixed infection by DTUs TcI/TcIV in PCR/RFLP assay. Of the two samples that were characterized as TcI/Z3 in the Mini exon and they were not further characterized by PCR-RFLP of H3/AluI, one was suggestive of infection by TcIII or TcV and another was shown to be infected by *T. cruzi* TcII, also suggesting a mixed infection (Table 3).

From 363 skin, spleen, and liver cultures, two skin cultures from a *D. albiventris* and an *O. cleberi* were positive. Similarity analysis of PCR products for the 18S rDNA gene confirmed the infection by, respectively, *T. rangeli* (MN381027-100% identity and 99% coverage) and *T. dionisii* (99.5% identity and 100% coverage), although, in the latter case, it was not possible to make a consensus sequence and parasite identification was based on only one sequence (Table 2).

Molecular diagnosis by kDNA-PCR resulted in four positive samples, all of them in liver fragments that were derived from *Calomys expulsus* (*N* = 1), *Oligoryzomys nigripes* (*N* = 1), and *G. agilis* (*N* = 2). These samples were submitted to PCR targeting HSP70 (234 base pairs) and only the two *G. agilis* samples were amplified, being identified as *L. (V.) braziliensis* (MN395479-99.6% identity and 96% coverage) e *L. (V.) guyanensis* (97.7% identity and 98% coverage). In this latter case, it was not possible to make a consensus sequence and parasite identification was based on only one sequence (Table 2).

### 2.3. Serological Diagnosis

Serology was performed in serum samples of 55 rodents (78.5% of the captured rodents), with 5.4% (*N* = 3) infected by *T. cruzi* and 3.6% (*N* = 2) for *Leishmania* spp. From the 74 captured marsupials, serology was performed on 68 of them (91.9%), presenting an infection rate of 14.7% (*N* = 10) and 2.9% (*N* = 2) for *T. cruzi* and *Leishmania* spp., respectively. Mixed infection was observed in 5.4% (*N* = 3) between rodents and 8.8% (*N* = 6) among marsupials, as shown in Table 4. Serological diagnosis provides information on a later infection when compared to molecular tests, which indicates that the host had become infected at some point in his life with the parasite, producing IgG class antibodies. This occurs despite the current presence of parasite’s DNA, which is unequivocally demonstrated by the molecular tests, indicating the presence of the parasite in that host.

## 3. Discussion

We demonstrated the small mammal fauna of the Limoeiro region and the most abundant species were *Gracilinanus agilis, Calomys expulsus, Oecomys cleberi,* and *Rhipidomys macrurus*. We observed *Trypanosoma cruzi* infection in *Didelphis albiventris, Oecomys cleberi, Rhipidomys macrurus,* and *G. agilis,* with the latter being the only species that displayed patent parasitemia and in which a distinguished diversity of DTUs was detected. We found other trypanosomatid species (*T. rangeli, T. dionisii, Leishmania braziliensis,* and *L. guyanensis*) in *D. albiventris, O. cleberi, C. expulsus,* and *G. agilis* showing a higher diversity of trypanosomatids in small mammals (*n* = 9) than that observed in wild canids (*n* = 2) of the same area [23,24].

On the ecology of the fauna studied, the higher number of small mammals captured in the dry seasons is probably due to greater availability of food in the rainy season, decreasing the effectiveness of baits and, consequently, capture probability [25,26]. Additionally, in the rainy season, most rodent and marsupial populations have few adults and many young, an age class generally less captured in bait traps [27,28]. Previous studies in the Cerrado, have detected a high density of rodents during the dry season [29,30], as registered at Limoeiro region, Cumari, most probably because some rodent species present breeding peaks in the rainy season, which leads to an increase in populations during the dry season [26,31]. The fourth expedition (wet and hot season) was marked by a capture rate that is similar to the first, probably because the environment could still be influenced by the end of the previous rainy season, which was prolonged in that year (2015). The significant increase in capture rate in the third expedition (dry and cold season) can also be explained by an atypical pluviometric index in the middle of 2014, which might have altered the natural reproductive dynamics of the extant small fauna.

Patent parasitemia by *T. cruzi* (flagellates in the fresh blood examination and DNA of parasite in hemocultures) were only observed in *G. agilis* (7/70 = 10%), which indicates their competence to infect vectors or other mammals, the last through predation [32]. DNA amplification was not possible for other three samples with flagellates in fresh blood examination. This can be explained, because it is a DNA-poor material (hemoculture sediment), which makes it difficult to recover.

*G. agilis* was infected by DTU TcI in both single and mixed infections with DTUs TcII, TcIII/TcIV, TcIV, and TcIII/TcV, corroborating that DTU TcI is the genotype most widely dispersed in nature [33,34]. Herein, we provided the first record of DTU TcII in *G. agilis* in the Cerrado biome, as well as of DTUs TcIII and TcIV in this species, showing that DTUs are more widespread both geographically and concerning their host ranges.

DTU TcII is also a widely distributed genotype and it has been found to infect several mammalian taxa in distinct habitats and biomes [35,36,37]. Although DTUs TcIII and TcIV are widely distributed and may infect a wide variety of mammals, they appear to occur at significantly lower rates, most likely because they are maintained in very low parasitemia in nature, which hampers their detection [23,32]. Although classically associated to armadillos [38,39], *T. cruzi* TcIII has also proved to be able to infect wild rodents, marsupials, carnivores, and artiodactyls, as well domestic dogs [5,23]. Previous long-term carnivore monitoring studies in the same area (Limoeiro Region, Cumari) registered two hoary foxes (*Lycalopex vetulus*) that were infected by *T. cruzi* DTU TcIII [22,23], which indicate the putative share of these DTUs between small mammals and canids of the same area. Finally, it is important to clarify that the finding of TcIII/TcV does not necessarily indicate mixed infection, once 18SrDNA sequences are not useful to separate hybrid genotypes, as is the case of TcV.

We used 18SrDNA target to confirm the results that were obtained by the mini-exon and this approach was useful to: (i) confirm four of the seven results observed in the mini-exon assay; (ii) clarify the DTU of one of the samples infected by Z3 genotype; and, (iii) demonstrate two mixed infections not detected in the mini-exon assay. Thus, 18S rDNA confirmed the infection by *T. cruzi* in all samples positive in the mini-exon assay, although it was not possible to obtain sufficient good quality sequences to make consensus in any of the samples, because it is a DNA-poor material (hemoculture sediment), resulting in sequences presenting not high coverage and identity values. Even if the mini-exon is no more considered as a reliable method for characterizing *T. cruzi* genotypes, it was useful in this case where the material investigated was poor in DNA, probably due to the higher number of copies in the DNA when compared to 18S rDNA target. We confirmed the majority of the DTUs for both targets, except for two samples: one positive for TCI/Z3 and another positive for TCI in the mini-exon, both being characterized as *T. cruzi* DTU TcII by 18S rDNA. This result might indicate a mixed infection by distinct *T. cruzi* genotypes. Moreover, a study using blood clots from these same samples detected *T. lainsoni* DNA (a parasite not detectable in the culture medium used) in the *G. agilis* that were infected by DTU TcI/Z3 [24] demonstrated a polyparasitism by *Trypanosoma* spp. in this specimen.

Our work also contributed to the first finding of *Trypanosoma rangeli* in skin cultures. However, the finding of *Trypanosoma dionisii* must be cautiously analyzed because identification was based on only one sequence of 360 nucleotides. However, once confirmed, this finding also represents the first encounter of this parasite in skin culture, reporting a broader spectrum of tissues that these parasites can be detected in mammals. In fact, in addition to *Trypanosoma caninum* detected in dog’s skin samples, the ability of other trypanosomatid species, such as *T. cruzi* and *T. brucei,* to infect the skin has already been demonstrated [40,41,42]. This is a factor that can explain the transmission of these parasites to vectors, even in the absence of detectable parasitemias. The findings of these two trypanosomatids in the skin suggest that they are: (i) most probably derived from skin microvessels; or, (ii) could be colonizing skin cells, since it is known that *T dionisii* can invade mammalian cells [43,44]. About *T. rangeli,* little is known of its life cycle in mammal hosts, but it has already been found colonizing an unorthodox site (bone marrow) [45,46], so that the colonization of both parasites in the skin cannot be ruled out. These findings may partially explain the transmission mechanism in the absence of high parasitemias. In addition to our findings, [24] evaluated infection by trypanosomatids in blood clots of 33 out of 70 *G. agilis* captured in the same area, identifying the infection by several species of *Trypanosoma*, including *T. dionisii* and *T. rangeli,* showing a larger spectrum of *Trypanosoma* species circulating in the area that were not found in canids [23,24].

Regarding *Leishmania* spp. infection in small mammals, there is only one previous record of infection by *L. (V.) braziliensis* in *G. agilis,* also in the Brazilian Cerrado (at Minas Gerais state) [47]. However, this is the first report of infection by this parasite in liver samples of this marsupial. Moreover, our work contributes to the finding of *L. (V.) guyanensis* in *G. agilis*, although other marsupial species have already been found to be infected by this parasite [48,49], which suggests that, in addition to other marsupials, *G. agilis* is also able to maintain a richness of trypanosomatids. Our register also represents a new geographical area for the distribution of *L. (V.) guyanensis,* never before registered to the Brazilian Cerrado.

In the Americas, more than 40 species of mammals were already described as hosts of *Leishmania* parasites [50]; however, only a minority of them act as reservoirs, being a source of infection to phlebotomine sand fly vectors; and, thereby, contributing to the dispersion of *Leishmania* [13,47]. On the other hand, regarding *Leishmania* infection through the predation of infected mammals, although the oral route has not yet been demonstrated, it is known that amastigotes are capable of infecting mammals, and this is a route that cannot yet be ruled out to occur in nature [51,52].

In addition to *G. agilis*, the Indirect Immunofluorescent Antibody Test (IFAT) detected infection by *T. cruzi* and *Leishmania* spp. in a broader range of local hosts than detected by the other methods, which complements the enzootic scenario by identifying infected individuals that were probably not infective to vectors at that moment (undetectable parasitemia) [32].

It is worth mentioning that most of the *G. agilis* that were infected by *T. cruzi* were captured in the understory stratum; however, these marsupials also circulate in the soil, which might favor contact with possible predators. A positive correlation was already demonstrated between *T. cruzi* infection rates and the proportion of mammals and insects in the diet of wild Carnivores [22], oral transmission being consistently suggested as the main mechanism of dispersion of the parasite among wild mammals [5,53]. In the specific case of *G. agilis*, this marsupial species represents less than 5% of the diet of wild canids (*C. thous, C. brachyurus,* and *L. vetulus*). This indicates that canids in the Limoeiro region do not frequently consume this marsupial [21], especially in the case of hoary fox, which was the species with the highest *T. cruzi* infection rates in the area. On the other hand, it is worth mentioning that one-time consumption of an infected animal is enough for the canid to become infected during its whole life, especially when considering that the lifetime of canids is about four or five times longer than that of small mammals, which increases the chances of being infected. On the other hand, rodents are consumed at much higher frequencies by the three canine species and they are constantly seen on pastures, areas widely used by these canids [21]. However, the intense heating that could favor death of small mammals inside the traps and the abundance of cattle were factors that impaired our sampling in this environment. For this reason, and knowing that wild canids rarely enter forested habitats, preferring edges of forest fragments and/or open areas of Cerrado [21], transects to capture small mammals were installed at the edges of fragments, or near fragments, and this might have increased the chance of capturing arboreal species, such as *G. agilis*.

*G. agilis* has biological and ecological characteristics that favor its exposure to the different transmission cycles of these parasites. This marsupial species presents an omnivorous diet that includes insects [54]; share refuges (such as tree or rocky holes) with triatomines or other insect vectors, besides exploring different forest strata. *G. agilis* is a representative of the Didelphidae family whose species are considered some of the oldest and most important trypanosomatid reservoirs, already considered as *T. cruzi* bioaccumulators, due to their ability to host an expressive diversity of trypanosome species [55]. Except for the rare cases of coprophagism and cleptophagism, triatomines become infected while feeding on an infected mammal, for example, an infected *G. agilis.* Besides the contaminative (*T. cruzi*) or inoculative *(Leishmania* spp.) route, the predation of infected small mammals or infected triatomines that acquired the infection by blood-sucking of infected small mammals seems to be the most likely route of *T. cruzi* infection for canids, revealing that small mammals and canids in that area are participating from the same transmission cycle of *T. cruzi.* Thus, the small mammals from Cumari are immersed in the transmission cycles of *T. cruzi* and *Leshmania* spp. and they share, at least, one *T. cruzi* DTU (TcIII) with wild canids from the same area. In addition, small mammals showed a higher diversity of *Trypanosoma* spp., as well as of *T. cruzi* genotypes, than observed in canids.

The complexity of the transmission cycles of *Trypanosoma cruzi* and *Leishmania* spp. in nature involves different components that change over time and space. Therefore, being able to study, in the same area, different elements of wildlife that maintain these parasites in nature provide us with complementary information that helps to have a broader understanding. Multidisciplinary work such as this has as its primary objective to contribute to the conservation of mammal species in anthropized areas, and their impacts on human and/or animal health

## 4. Materials and Methods

### 4.1. Study Area

**Figure 1:**
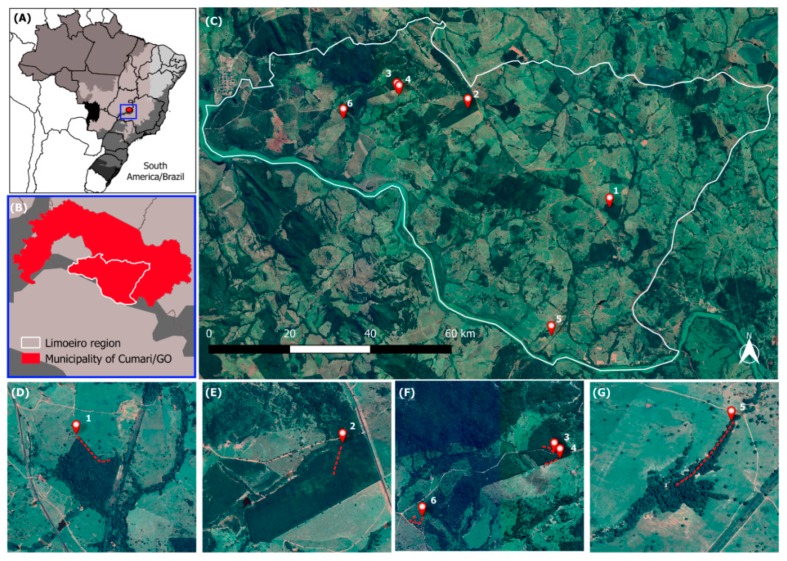
Study area located in the Limoeiro region, municipality of Cumari, southeast of Goiás State, Brazil (**A**–**C**), formed by private cattle farms with forest remnants where small mammals were captured. Figures (**D**–**G**) show the arrangement of the six transects (1 to 6) at the edges of forest remnants.

The study area (Figure 1) comprises private cattle farms of the Limoeiro region, in the municipality of Cumari (18°22.02′ S, 48°5.48′ W), southeast of Goiás State, Brazil. Most of the area (75%) has been altered by human activities and it is mostly covered by exotic pasture (*Urochloa* sp.). Patches of original vegetation (Atlantic Forest and Cerrado), such as gallery and semi-decidous forests, represent the remaining 25% [56]. The climate in the region has two well-marked seasons, one hot and wet and another cold and dry [57]. We focused on the end of the seasons, also considering rainy periods when they exceed the wet season, to define the collection period. Captures were performed in: (i) August 2013; (ii) April 2014; (iii) September 2014; and, (iv) June 2015. Mean annual temperature and rainfall vary between 22–25 °C and 1600–1800 mm, respectively (data from Center for Weather Forecast and Climate Studies (CPTEC), National Institute for Space Research (INPE)).

### 4.2. Small Wild Mammal Capture and Identification

Small mammals were captured in six transects of 20–40 trap points each, while using live traps (Tomahawk Live Traps, Tomahawk, WI, USA and Sherman-H. B. Sherman Traps, Tallahassee, FL, USA). The traps were disposed at 10 m intervals during five consecutive nights, alternately distributed both in the ground and understory strata, and baited with a mixture of banana, peanut butter, oat, and bacon or sardines [44]. The transects were placed in areas of riparian and semi-deciduous forests known to be partially used by wild canids (Figure 1). The points of capture of the small mammals were georeferenced through the Global Positioning System receiver (GPS-Montana 650 Garmin^®^), with World Geodetic System 84 (WGS 84) as the geodetic reference. The total capture effort was 1300 trap-nights/expedition (totaling 5200 trap-nights during four expeditions). The ratio between sampling effort in the understory and ground strata was 1600/3600 traps, respectively, corresponding to 80 traps in the understory and 180 traps on the ground. Whenever a small mammal was captured, the trap was identified, placed in a plastic bag, and then transported to the field laboratory.

The identification of specimens was based on external and cranial morphological characters and on karyological analyses [58]. Euthanized mammals were deposited as vouchers specimens in the Mammal Collection of the National Museum from Rio de Janeiro Federal University (UFRJ), Brazil.

#### Field Procedures

The captured small mammals were anesthetized with an intramuscular injection of Ketamine (10–30 mg/kg) associated with: (i) acepromazine (5–10mg/kg) for rodents (9:1); or, (ii) xylazine (2 mg/kg) for marsupials (1:1). Blood samples were collected by cardiac puncture for parasitological and serological analysis, as follows: fresh blood examination, hemoculture, and centrifugation to obtain serum.

The fresh blood examination was carried out with a drop of approximately 5 µL of blood between slide and coverslip. For hemoculture, it was inoculated 0.6–0.8 mL of blood from each animal in two tubes (0.3–0.4 mL each) containing Novy-Neal-Nicolle (NNN) medium with Liver Infusion Tryptose (LIT) overlay [22]. Serum was stocked at −20 °C for serology assay after blood centrifugation. Priority was given to hemoculture when insufficient blood volume was obtained.

All of the animals were euthanized while using Potassium Chloride 19.1% by intracardiac route. Samples of skin, spleen, and liver were collected in tubes containing absolute ethanol for Polymerase Chain Reaction (PCR) or sterile saline (sodium chloride-NaCl at 58.44 g/mol) with antibiotics and antifungals (10 mg streptomycin, 25 μL amphotericin B, and 10,000 IU penicillin per mL, Sigma^®^ commercial solution) for culture. After being stored at 4 °C for 24 h in this solution, tissue fragments were transferred to culture tubes containing NNN medium and Schneider liquid medium [59].

### 4.3. Infection Diagnosis Procedures

Fresh blood examinations were analyzed in the field laboratory while using an optical microscope at 400× magnification. Hemocultures were examined every 15 days up to five months [22], while tissue cultures were examined every four days up to two months. Positive tissue cultures and/or hemocultures that were derived from mammals positive in the fresh blood examination (even when negative during the hemoculture examination) were centrifuged for sediment formation and then incubated with proteinase K and Sodium Dodecyl Sulfate (SDS). In this case, the genomic DNA was extracted with the standard phenol-chloroform method [60].

A serological survey for the detection of anti-*T.cruzi* and anti-*Leishmania* IgG antibodies was performed while using an Indirect Immunofluorescent Antibody Test (IFAT) [61]. Antigens used in the reaction to *T. cruzi* were an equal mixture of parasites that were derived from the strains M000/BR/1974/F90 (TcI) and MHOM/BR/1950/Y (TcII), obtained from the Collection of Trypanosoma from Wild and Domestic Mammals and Vectors (COLTRYP) of the Oswaldo Cruz Foundation (FIOCRUZ - Rio de Janeiro-RJ/Brazil). Antigens used in *Leishmania* surveys were an equal mixture from the stains MHOM/BR/1975/M2903 (*L. braziliensis*-IOC/L566) and MHOM/BR/1974/PP75 (*L. infantum*-IOC/L579) obtained from the Leishmania Collection from Oswaldo Cruz Institute (CLIOC-FIOCRUZ/RJ).

Serum of Cricetidae rodents were tested with a commercial anti-hamster IgG conjugate (FITC, Sigma-Aldrich^®^, St Louis, MO, USA), whereas marsupials’ sera were tested while using intermediary anti-IgG antibodies for *Didelphis aurita* in rabbits [62]. The reaction was revealed while using an anti-rabbit IgG antibody that was conjugated with fluorescein (Sigma, St Louis, MO, USA). Cutoff values adopted were 1:40 for marsupials and 1:10 for rodents [63].

### 4.4. Molecular Diagnosis and Characterization

Two techniques were used for the molecular characterization of flagellates that were derived from hemocultures. The first one was the Multiplex PCR to amplify the non-transcribed spacer of the mini-exon gene [64]. When positive, the samples were then submitted to the Nested PCR of the variable region of the trypanosome 18S rDNA gene [65,66]. Only the last technique was applied to positive skin cultures.

Multiplex PCR were employed, aiming to amplify the non-transcribed spacer of mini-exon gene for the identification of subtypes TCI (Discrete Typing Units-DTU TcI-200 pb), TCII (DTUs TcII/TcIV/TcV-250 pb), Z3 (DTUs TcIII/TcIV-150 pb), and *Trypanosoma rangeli* (100 pb) [67], as well as mixed infections. Positive samples for Z3 were further amplified by PCR for the histone 3 (H3) gene [68], followed by Restriction Fragment Length Polymorphism (RFLP) analysis after digestion by the AluI enzyme for discrimination of TcIII and TcIV [44].

The nested PCR of the variable region of the trypanosome’s 18S rDNA gene consists of two rounds: one that amplifies a larger region of the target, using 16 pmol/μL of the primers: TRY927F5′ GAAACAAGAAACACGGGAG′ and TRY927R-5′CTACTGGGCAGCTTGGA3′, and another where the amplified product of this first round is diluted 1:5 in sterile deionized water and employed in the second round with 16 pmol/μl of the internal primers: SSU561F 5′TGGGATAACAAAGGAGCA3′ and SSU561R 5′CTGAGACTGTAACCTCAAAGC3′ [65,66].

Electrophoresis of PCR products were carried out in a 2% agarose gel, stained with GelRed-Biotium, and then visualized under UV light. Each reaction included sterile distilled water instead of DNA as a negative control and positive control samples from *T. cruzi* strains that represent the DTUs [22,69].

Tissue fragments that were collected in absolute ethanol were re-hydrated with Nuclease-free water and the DNA extraction was carried out while using the Wizard Genomic DNA Purification Kit (Promega, Madison, USA), according to the manufacturer’s recommendations. PCR were performed using the pureTaq Ready-To-Go PCR beads (Amersham Biosciences, Buckinghamshire, UK), and primers directed to the conserved region of the *Leishmania*-kDNA mini circle were: forward: 5′-GGGAGGGGCGTTCTGCGAA-3′ and reverse: 5′-GGCCCACTATATTACACCAACCCC-3′ [70,71]. The positive and negative controls were derived from spleen and liver fragments from infected (*Leishmania* braziliensis-IOC-L2483) and non-infected hamsters that were provided by the animal facilities of the FIOCRUZ/RJ. The PCR products were visualized after electrophoresis on 8% polyacrylamide gel and silver stained while using a specific kit (DNA Silver Staining, GE Healthcare). Positive samples were submitted to another PCR directed to the variable region of the gene coding for the Heat Shock 70 protein (HSP70), a fragment of 234 base pairs, using the following primers (5′-GGACGAGATCGAGCGCATGGT-3′) and (5′-TCCTTCGACGCCTCCTGGTTG-3′) [70,72]. Samples from this latter target were submitted to purification using DNA purification kit (GE HealthCare Life Sciences, UK) and following the manufacturer’s instructions.

The sequencing reaction was performed while using the BigDye Terminator v3.1 kit (Applied Biosystems, USA) and then applied to the ABI3730 DNA analyzer automatic sequencer (Applied) in the Sequencing Platform (RPT01A) of the FIOCRUZ/RJ. The editing and construction of the sequences were performed with the SeqMan-DNA Star Program and the consensus sequences were aligned and edited with the BioEdit Version v7.1.11. Sequence analysis for species identification was performed by similarity that was obtained through Basic Local Alignment Search Tool (BLAST) algorithm against sequences available on GenBank (National Center for Biotechnology Information, NCBI).

### 4.5. Statistical Analysis

Statistical comparisons between the richness and abundance of small mammal species, richness and abundance of species per season, and capture success per seasons were performed using binomial tests on BioEstat 5.0 software (Mamirauá Institute/ Manaus, Amazonas state, Brazil), and a significance level of 5% was adopted.

### 4.6. Ethics Statement

The handling of the animals was performed according to the biosafety standards established by the Biosafety Commission of the Oswaldo Cruz Institute, IOC/FIOCRUZ following protocols that were approved by the Oswaldo Cruz Foundation’s Ethics Committee on Animal Use (LW81/12). Capture and euthanasia of wild animals was licensed under SISBIO (13373-1).

### 4.7. Map Construction

For the map construction, the study area and the trailing points of the captured small mammals were visualized in a Geographic Information System (GIS) in the Quantum GIS software version 2.18, using the continental, national, and municipal boundaries of the study area, extracted from the open access cartographic base of Brazilian Institute of Geography and Statistics (IBGE). Google Earth Satellite images (QGIS QuickMapServices plugin) were also used.

## Figures and Tables

**Table 1:** Small mammals captured at Limoeiro Region, Municipality of Cumari, Goiás, Brazil, between 2013 and 2015.

Order	Species	Expedition			
		1st Dry and Cold	2nd Wet and Hot	3rd Dry and Cold	4th Wet and Hot
Rodentia	*Calomys tener*	6	-	2	-
	*Calomys expulsus*	6	-	24	-
	*Rhipidomys macrurus*	2	2	3	2
	*Hylaeamys megacephalus*	1	1	2	-
	*Oligoryzomys mattogrossae*	1	-	-	-
	*Oecomys cleberi*	1	1	7	6
	*Necromys lasiurus*	-	-	1	-
	*Oligoryzomys nigripes*	-	-	2	-
	Total rodents	17	4	41	8
Didelphimorphia	*Gracilinanus agilis*	17	1	30	22
	*Didelphis albiventris*	-	2	1	1
	Total marsupials	17	3	31	23
Total of captures (*n* = 144)/expedition (*n* = 4)		34	7	72	31

**Table 2:** Parasitological and molecular diagnosis of captured small mammals at Cumari, Goiás, Brazil.

Expeditions/Captured Animals		1st Expedition/34	2nd Expedition/7	3rd Expedition/72		4th Expedition/31	
Positive parasitological/molecular diagnosis	Fresh blood examination	3	1 *	3		3	
	Hemoculture	-	1 *	-		-	
	Skin, spleen or liver culture	-	-	2 SKINS		-	
	Skin, spleen or liver in ethanol for kDNA-PCR	-	-	-		4 LIVERS	
	Parasite identification	TcI (*n* = 2) and TcI/Z3 (Discrete Typing Unit - DTU TcIII/IV) (*n* = 1)	TcI (*n* = 1)	Blood: TcI/TcIV (*n* = 2) TcI/Z3 (DTU TcIII/IV) (*n* = 1)	Skin: *T. rangeli* (*n* = 1) *T. dionisii* (*n* = 1) **	Blood: Not amplified (*n* = 3)	Liver: *Leishmania* spp. (*n* = 2) *L. braziliensis* (*n* = 1) *L. guyanensis* (*n* = 1) **
	Mammal species	*G. agilis* (*n* = 3)	*G. agilis* (*n* = 1)	*G. agilis* (*n* = 3)	*Didelphis albiventris* (*T. rangeli*) *Oecomys cleberi* (*T. dionisii*)	*G. agilis* (*n* = 3)	*C. expulsus* and *O. nigripes* (*n* = 2 *Leishmania* spp.) *G. agilis* (*L. braziliensis* and *L. guyanensis*)

* Same mammal host. ** It was not possible to make a consensus and the identification of parasite was based only on the reverse sequence.

**Table 3:** Comparison of results between mini-exon and 18S rDNA molecular targets, for characterization of *Trypanosoma cruzi* infection from hemoculture sediments whose fresh blood examination was positive.

ID of Sample	Mini Exon/Restriction Fragment Length Polymorphism Results	Similarity to genBank Sequences by the 18S rDNA Target (Coverage/Identity—%) *
LBCE 15978	DTU TcI	*T cruzi* TcI-Reverse Stranded: 96/78.86%
LBCE 15979	DTU TcI/Z3	*T cruzi* TcIII/TcV-Reverse Stranded: 100/83.65%
LBCE 15980	DTU TcI	*T. cruzi* TcI-Forward Stranded: 95/86.11% *T. cruzi* TcI-Reverse Stranded: 99/95.64%
LBCE 18574	DTU TcI	*T cruzi* TcII-Foward Stranded: 97/76.38%
LBCE 18583 **	DTU TcI/TcIV	*T. cruzi* TcIV-Forward Stranded: 100/68.03% *T. cruzi* TcIV-Reverse Stranded: 98/73.19%
LBCE 18584 **	DTUTcI/TcIV	*T. cruzi* TcIV-Forward Stranded: 99/72.20% *T. cruzi* TcIV-Reverse Stranded: 93/74.26%
LBCE 18586 **	DTU TcI/Z3	*T cruzi* TcII-Reverse Stranded: 100/100%

* Note: The sequences were analyzed in the BioEdit (Atlanta, GA, USA) and SeqMan (Madison, WI, USA) programs and compared to GenBank sequences only through the Somewhat Similar Sequences (Blastn) tool (National Center for Biotechnology Information, Bethesda, MD, USA), except for LBCE 15979 and LBCE 15980 samples, that it was possible to obtain results from the Megablast (Highly similar sequences). ** These animals were also positive for *Trypanosoma lainsoni* in blood clot samples [24].

**Table 4:** Percentage of positivity in the Indirect Immunofluorescent Antibody Test (IFAT) test for *Trypanosoma cruzi, Leishmania* spp. and mixed infection in small mammals (rodents and marsupials) per expedition.

Infection by	1st Expedition		2nd Expedition		3rd Expedition		4th Expedition	
	Rodents	Marsupials	Rodents	Marsupials	Rodents	Marsupials	Rodents	Marsupials
*T. cruzi* (Positive)	1/7 (14.3%)	4/14 (28.6%)	1/4 (25%)	2/3 (66.7%)	1/37 (2.7%)	4/30 (13.3%)	0/7 (0%)	0/20 (0%)
*Leishmania* spp. (Positive)	0/7 (0%)	1/14 (7.1%)	0/4 (0%)	0/3 (0%)	2/37 (5.4%)	1/30 (3.3%)	0/7 (0%)	0/20 (0%)
Mixed infection	2/7 (28.6%)	3/14 (21.4%)	0/4 (0%)	1/3 (33.3%)	1/37 (2.7%)	2/30 (6.6%)	0/7 (0%)	0/20 (0%)
